# Evaluation of the Prevalence, Trends, and Correlates of Low Back Pain in India: A Retrospective Cross-Sectional Study

**DOI:** 10.7759/cureus.83518

**Published:** 2025-05-05

**Authors:** Sunil H Shetty, Amarnath S S., Arnab Karmakar, Monjori Mitra, Nishikant Madkholkar, Roshan Pawar, Akhilesh Sharma

**Affiliations:** 1 Orthopedics, Dr. D. Y. Patil Medical College, Hospital, and Research Centre, Mumbai, IND; 2 Trauma and Orthopedics, Trinity Central Hospital, Bengaluru, IND; 3 Orthopedics, Institute of Post-Graduate Medical Education and Research and Seth Sukhlal Karnani Memorial Hospital, Kolkata, IND; 4 Pediatrics, Institute of Child Health, Kolkata, Kolkata, IND; 5 Medical Affairs, Alkem Laboratories Private Limited, Mumbai, IND

**Keywords:** adults aged 18 and older, body mass index (bmi), calcium therapy, comorbidity profiles, non-steroidal anti-inflammatory drugs (nsaids), real-world evidence

## Abstract

Background: Low back pain (LBP) is a leading cause of disability worldwide, including a significant portion of the Indian population; however, comprehensive epidemiological data are lacking. This study aimed to explore the prevalence, risk factors, and prescribing patterns for LBP in the Indian population.

Methods: This cross-sectional, retrospective study (CTRI/2025/01/079359 (registered on: January 24, 2025)) analyzed anonymized electronic medical record (EMR) data of 16866 patients from 76 private centers in 18 states across India, diagnosed with LBP between 2019 and 2023. The primary outcomes include the assessment of the prevalence of LBP across demographic and clinical subgroups, the distribution of treatment practices among various medical specialties, and the utilization of medication classes such as nonsteroidal anti-inflammatory drugs (NSAIDs) and calcium supplements. Secondary outcomes include the assessment of the prevalence of LBP categorized by sex, BMI, age group, and comorbidities, as well as the proportion of patients with LBP treated by physicians of different medical specialties. Anthropometric parameters, including weight, height, and body mass index (BMI), were assessed. The prevalence of LBP, associated symptoms, comorbidities, and prescribing trends were examined using statistical analyses.

Results: Among 16866 patients diagnosed with LBP, the prevalence of LBP fluctuated across the five-year study period (2019-2023). From 3767 cases (22.33%) in 2019, prevalence declined sharply to 2762 cases (16.38%) in 2020 (χ² = 154.7, p < 0.001), followed by a significant increase to 3272 cases (19.40%) in 2021 (χ² = 43.11, p < 0.001 compared to 2020). Prevalence remained statistically stable in 2022 at 3248 cases (19.26%; χ² = 0.09, p = 0.766 compared to 2021), before rising significantly to 3817 cases (22.63%) in 2023 (χ² = 45.83, p < 0.001 compared to 2022). Notably, while the 2023 prevalence represented the highest point in the study period, it was not statistically different from the 2019 baseline (χ² = 0.33, p = 0.566). Females exhibited a significantly higher prevalence of LBP compared to males (11420 (67.71%) vs. 5546 (32.29%), p < 0.001). Additionally, 10328 (61.24%) patients were classified as overweight or obese. Among patients with comorbidities, 3870 (22.95%) patients presented with gastrointestinal disorders, 2763 (16.38%) had orthopedic conditions, and 2226 (13.20%) were diagnosed with hypertension. These conditions were identified as significant predictors of LBP. Calcium supplements were utilized by 4479 (26.56%) patients, NSAIDs by 4350 (25.79%), and NSAID-muscle relaxant combinations by 1342 (7.96%) patients, with a limited use of opioids in 633 (3.75%) patients.

Conclusion: This pan-Indian cross-sectional study highlighted LBP as a major health burden affecting anthropometric parameters. Key risk factors included female gender, young adulthood, and high BMI, emphasizing the need for targeted management strategies.

## Introduction

Globally, low back pain (LBP) remains the leading cause of disability. In 2020, LBP accounted for 64.9 million disability-adjusted life years (DALYs) [1]. LBP affects 30.0% of people in high-income countries (4715 out of 15716 individuals) compared to 18.0% in low-income countries (8406 out of 46700 individuals) [2]. The estimated prevalence of LBP in India varies significantly, ranging from 105 of 250 (42%) [3] to 251 of 301 (83%) [4], primarily reflecting methodological differences in assessment timeframes, measurement instruments (Numerical Pain Scale [3] vs. Nordic Musculoskeletal Questionnaire [4]), sampling techniques, and regional variations between northern and southern India, thus highlighting the need for standardized approaches in LBP epidemiological research. Research indicates that approximately 60% of the population has experienced LBP at least once in their lifetime [3,4]. This high prevalence underscores the importance of addressing LBP as a public health concern and highlights the need for effective interventions and management strategies within the healthcare system. LBP is ranked as the second most common reason for years lived with disability (YLD) after iron deficiency anemia in India [5]. In lower-income and lower-middle-income countries, the burden of LBP showed a 50% increase in the last 20 years [6].

Despite numerous studies documenting the global prevalence of LBP and its associated risk factors, there remains a significant paucity of epidemiological data regarding LBP within the Indian population. Existing reviews on clinical progress and pain intensity are limited in scope, relying on restricted data and participants. There is a need to synthesize existing knowledge about LBP in the adult population, particularly focusing on anthropometric characteristics (such as body mass index (BMI), weight distribution, and height) as potential risk factors. Elevated BMI and abnormal weight distribution may increase mechanical stress on the lumbar spine, alter biomechanics, and contribute to inflammatory processes, all of which could enhance prevention and treatment strategies [7]. This could enhance prevention and treatment strategies. While studies have identified various primary risk factors, they often interact as confounding variables with differing significance. Research has pinpointed vulnerable age demographics, but investigations into the relationship between mortality rates in these populations and external stressors are limited [8]. Epidemiological analyses have largely concentrated on adult populations, and studies have been performed to envisage the recovery of LBP [9]. Detecting the impact of typical conceivable risk factors for LBP among the older population is more difficult, as additional contributory or aggravating determinants will increasingly obscure the clinical scenario.

Studies on LBP patients reveal common symptom patterns, including localized pain, radiculopathy, and sciatica [10]. Additionally, orthopedic conditions like degenerative disc disease and arthritis occur in a considerable proportion of LBP patients [11]. While lower prevalence rates of hypertension and psychological comorbidities are noted in the data, they may suggest underreporting or specific traits of the patient population.

The study aimed to investigate the relationship between the anthropometric parameters (weight (kg), height (mt), and BMI (kg/m²)) in Indian patients with LBP. It highlighted the lack of community-based studies on LBP prevalence in India. The primary goal was to conduct a comprehensive cross-sectional survey with a representative sample of Indian adults to estimate LBP prevalence, identify risk factors, and gather current prescribing patterns. Analyzing electronic medical records (EMRs) will help inform public health strategies to alleviate the burden of LBP.

## Materials and methods

Study design

This cross-sectional, retrospective, multicenter observational (CTRI/2025/01/079359 (registered on: January 24, 2025)) study examined anonymized patient profiles of individuals with LBP in India and the treatment strategies employed for approximately five years (January 1, 2019, to December 31, 2023). Data were sourced from EMRs across various specialties from 85 centers, encompassing 1634 physicians from 76 private centers in 18 states throughout India through purposive sampling. Of these 76 private centers, 37 were in urban areas, while 39 were in rural regions. Among the 1634 physicians, 1355 practiced in urban and 279 in rural centers. A comprehensive EMR validation was implemented through sequential quality assurance steps. Initial screening excluded records with incomplete demographic data or ambiguous LBP diagnosis, followed by data validation to ensure consistency in coded data across centers. The study protocol was approved by the Royal Pune Independent Ethics Committee (RPIEC081124; dated: November 8, 2024).

Inclusion and exclusion criteria

The inclusion criteria for this study consist of male and female patients aged 18 years or older who have been diagnosed with LBP by their treating physician. Eligible patients must have EMRs dated between January 1, 2019, and December 31, 2023. Conversely, patients will be excluded if their records contain missing or incomplete information related to demographics, clinical characteristics, or endpoint analysis.

Study outcomes

The primary outcomes include the assessment of the prevalence of LBP across demographic and clinical subgroups, the distribution of treatment practices among various medical specialties, and the utilization of medication classes such as nonsteroidal anti-inflammatory drugs (NSAIDs) and calcium supplements. Secondary outcomes include the assessment of the prevalence of LBP categorized by sex, BMI, age group, and comorbidities, as well as the proportion of patients with LBP treated by physicians of different medical specialties.

Anthropometric measurements of parameters

Three anthropometric measurements were obtained for all patients: weight (kg), height (mt), and BMI (kg/m²). BMI was then calculated for both male and female patients using these values. The doctors categorized the initial diagnoses and observed conditions into three groups: underlying health issues, potential causes of lower back pain, and symptoms associated with the pain. This approach helped them better understand the complex nature of each patient's case. The treatment options reported as preferred regimens were grouped into several categories: NSAIDs, fixed-dose combinations of NSAIDs and muscle relaxants, vitamins and minerals, hormones, calcium supplements, and opioids. The analysis also highlighted the medical specialties of the healthcare providers treating this condition.

Statistical analysis

Data were retrieved, collated, and cleaned in MS Excel (Microsoft Corporation, Redmond, USA) from EMRs. All statistical analyses were done using IBM SPSS Statistics for Windows, Version 29 (Released 2021; IBM Corp., Armonk, New York, USA). Available data for planned endpoints were used for statistical analysis. All study variables were summarized using descriptive statistics. Continuous variables were reported as mean ± standard deviation (SD), median, minimum, maximum, or 95% confidence interval, as appropriate. Frequency and proportions/percentages were reported for categorical variables, and the chi-square test was used for comparison of the same. The p-values less than 0.05 were considered statistically significant.

## Results

Patient characteristics

**Figure 1:**
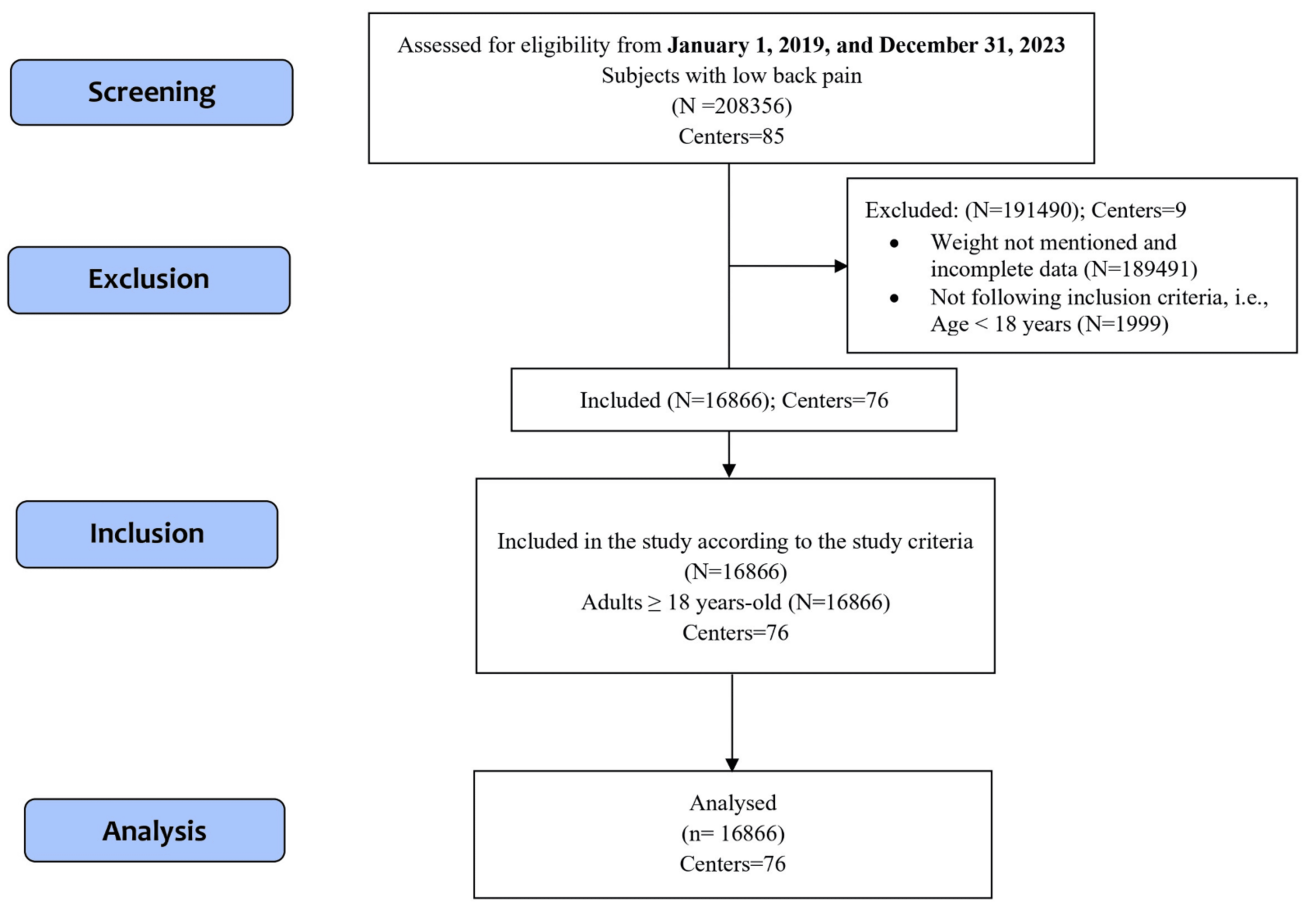
STROBE diagram showing selection of patients diagnosed with low back pain STROBE: Strengthening the Reporting of Observational studies in Epidemiology

**Figure 2:**
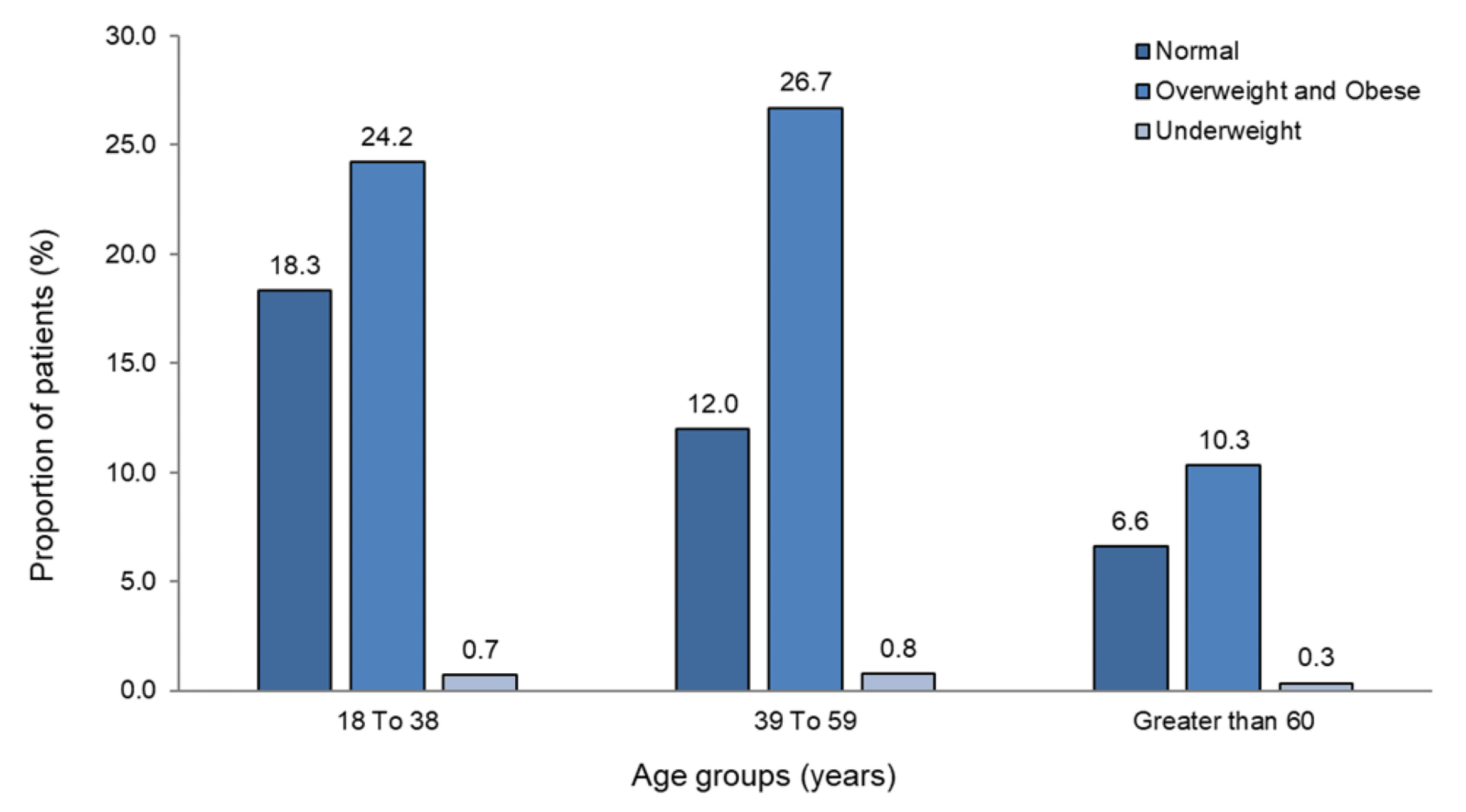
Distribution of BMI categories in each age group

Among 208356 patients diagnosed with LBP from 85 centers, 16866 from 76 centers in 18 states across India were screened for analysis based on the eligibility criteria as depicted in Figure 1. The mean BMI of 16866 patients was 27.00 (SD 5.36) kg/m². Among 16866 patients with LBP, 10328 (61.24%) are overweight or obese, 6228 (36.93%) have a normal BMI, and 310 (1.84%) are underweight. The differences between these categories are statistically significant (p < 0.001) (Table 1). The distribution of BMI categories across age groups is depicted in Figure 2. Regarding comorbidities, gastrointestinal issues were most common in 3870 (23.95%) patients, followed by orthopedic conditions in 2763 (16.4%) and hypertension in 2226 (13.2%) patients (Table 1).

**Table 1:** Demographic and clinical characteristics of the patients p < 0.05 is considered statistically significant; ^#^taken from the National Family Health Survey 2015-16 (NFHS-4) data BMI (kg/m^2^): Underweight: less than 18.5; Normal weight: 18.5 to 24.9; Overweight: 25.0 to 29.9; Obese: greater than 30

Parameters	Subgroups	N (%)	Mean (SD)	χ^2^	p-value
Total number of patients	-	16866	-	-	-
Gender	Male	5546 (32.29%)	-	-	<0.001
	Female	11420 (67.71%)	-	4090.3	
Weight, kg	-	-	65.39 (12.95)	-	-
Height^#^, mt	-	-	1.56 (0.05)	-	-
BMI, kg/m^2^	-	-	27.00 (5.36)	-	-
BMI range, kg/m^2^	Normal	6228 (36.93%)	-	-	-
	Underweight	310 (1.84)	-	6642.4	<0.001
	Overweight and obese	10328 (61.24%)	-	1993.1	<0.001
Age, years	-	-	43.09 (15.01)	-	-
Age group	18-38	7294 (43.25%)	-	2692.3	<0.001
	39-59	6657 (39.47%)	-	2041.4	<0.001
	≥60	2915 (17.28%)	-	-	-
Co-morbidities	Hypertension	2226 (13.20%)	-	-	-
	Diabetes mellitus	1807 (10.71%)	-	-	-
	Renal disorders	366 (2.17%)	-	-	-
	GI disorders	3870 (22.95%)	-	-	-
	Orthopedic disorders	2763 (16.38%)	-	-	-
	Gynecological disorders	812 (4.81%)	-	-	-

Prevalence and trends of LBP

**Figure 3:**
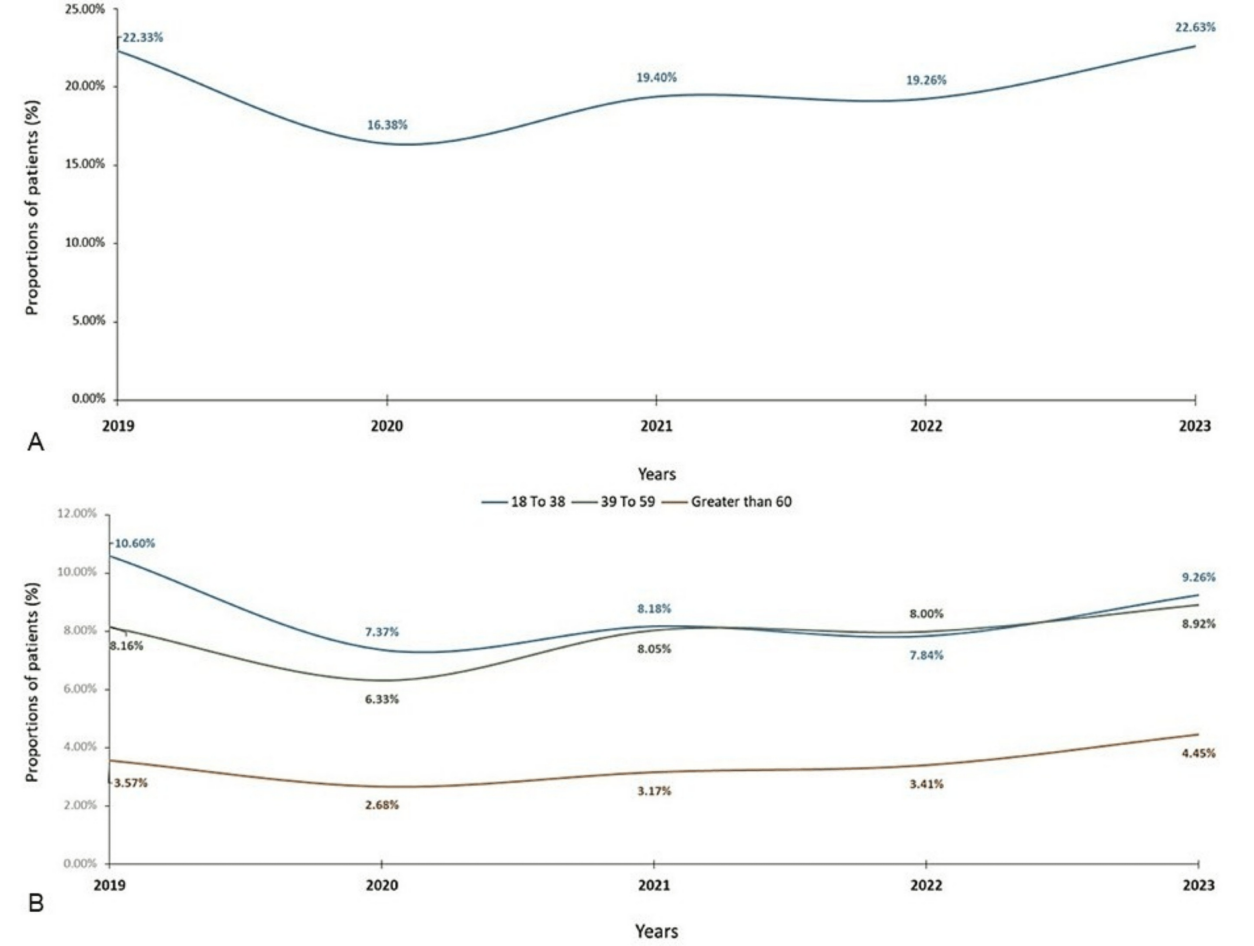
(A) Overall prevalence and (B) age group-wise prevalence of LBP over five years from 2019 to 2023 LBP: low back pain

The mean (SD) age of 16866 patients diagnosed with LBP was 43.09 (15.01) years. Of 16866 patients, the prevalence of LBP varied significantly from 2019 to 2023 (Figure 3A). It was 3767 cases (22.33%) in 2019, then dropped to 2762 cases (16.38%) in 2020 (χ² = 154.7, p < 0.001). It significantly increased to 3272 cases (19.40%) in 2021 compared to 2020 (χ² = 43.11, p < 0.001 compared to 2020). Prevalence remained statistically stable in 2022 at 3248 cases (19.26%; χ² = 0.09, p = 0.766 compared to 2021), before rising significantly to 3817 cases (22.63%) in 2023 (χ² = 45.83, p < 0.001 compared to 2022). Notably, while the 2023 prevalence represented the highest point in the study period, it was not statistically different from the 2019 baseline (χ² = 0.33, p = 0.566) (Table 2). LBP prevalence showed a decline from 2019 to 2020 across all age groups (Figure 3B). After 2020, there was a gradual increase in LBP prevalence, particularly in individuals over 60. The 18-38 age group had the highest prevalence of LBP, except in 2020. The 39-59 age group had a lower prevalence of LBP. Females showed a higher prevalence of LBP for the study period, with 11420 cases (67.71%), compared to 5446 cases (32.29%) in males (p < 0.001).

**Table 2:** Year-wise prevalence of low back pain from 2019 to 2023 p < 0.05 is considered statistically significant

Year	Cases (N)	Prevalence (%)	Year comparison	χ^2^	p-value
2019	3767	0.2233	2019-2020	154.7	<0.001
2020	2762	0.1638	2020-2021	43.1	<0.001
2021	3272	0.194	2021-2022	0.1	0.766
2022	3248	0.1926	2022-2023	45.8	<0.001
2023	3817	0.2263	2019-2023	0.3	0.566

Prescribers for LBP

**Figure 4:**
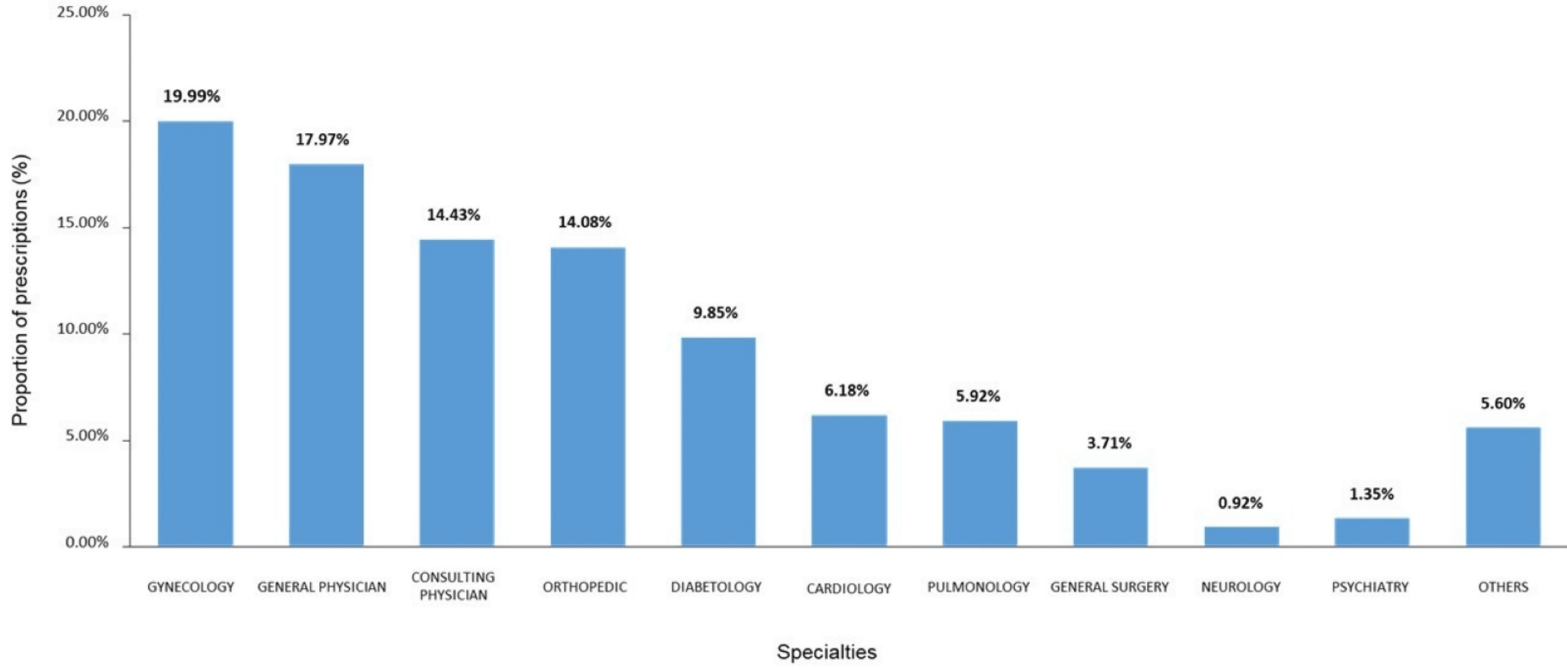
Specialty-wise patient visits presented to the healthcare physicians The total number of subjects (N) = 16866

Gynecologists were the most frequent prescribers for LBP, accounting for approximately 3372 (20%) prescriptions. This was followed by 3030 (17.97%) prescriptions by general physicians, 2434 (14.43%) by consulting physicians, 2375 (14.08%) by orthopedic specialists, 1661 (9.85%) by diabetologists, 1042 (6.18%) by cardiologists, and 626 (3.71%) by general surgeons. Other medical specialties collectively accounted for 2326 (13.72%) LBP prescriptions (Figure 4).

Pharmacologic and non-pharmacologic prescribing patterns

**Figure 5:**
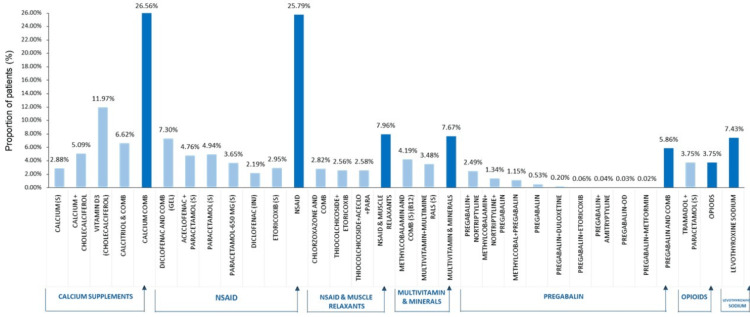
Prescription pattern and medication utilization distribution among LBP patients over five years (2019-2023) LBP: low back pain; NSAID: nonsteroidal anti-inflammatory drug

According to Figure 5, calcium combinations are the most frequently used therapy in 4479 (26.56%) cases, followed closely by NSAIDs in 4349 (25.79%) cases. NSAIDs combined with muscle relaxants were prescribed in 1342 (7.96%) patients, while multivitamins and mineral supplements accounted for 1293 (7.67%) prescriptions. Other notable interventions include 1253 (7.43%) prescriptions for levothyroxine, 988 (5.86%) for pregabalin combinations, and 632 (3.75%) for opioids.

## Discussion

This comprehensive study provides valuable epidemiological insights into the prevalence, risk factors, and management of LBP in the Indian population. The progression patterns of LBP, whether transient, recurrent, or chronic, remain unclear and require further investigation. Identifying early symptoms is essential for creating effective prevention strategies. Recent studies have found various risk factors for LBP, including physical traits, psychological issues, lifestyle choices, work demands, social situations, and genetic factors [12-14].

This study is the first community-oriented cross-sectional analysis of LBP prevalence in the pan-Indian population of 16866 patients, while earlier studies on Indian LBP patients examined trends among Indian physiotherapists regarding assessment and management strategies, compared specific treatment approaches, or investigated biopsychosocial factors in specific urban or rural communities [3,4,15,16]. Our findings reveal a striking gender disparity in LBP prevalence, with females exhibiting a higher lifetime prevalence of LBP compared to men (11420 (67.71%) vs. 5546 (32.29%)), consistent with a previous study [17]. Several biopsychosocial mechanisms have been proposed to explain this gender difference, including fluctuations in sex hormones, genetic markers, and variations in the endogenous opioid system [15]. Repetitive physical activities by females, especially household chores, are associated with increased reporting of back pain, which is generally linked to spine issues like microfractures. Both biological factors and lifestyle choices contribute to the gender gap in LBP [18].

The age-specific analysis of LBP prevalence provides further insights. The consistently higher prevalence among the younger age group (18-38 years) compared to the middle-aged (39-59 years) and older (>60 years) cohorts is an interesting finding that aligns with an earlier study [8]. This observation may be attributed to increased physical demands, lack of ergonomic awareness, and higher rates of occupational injuries in younger adults [19]. Our data on the prevalence of LBP are comparatively lower than that documented earlier for LBP or chronic back pain: 871 (57%) cases were reported in a previous community-based population in Northern India [20], and 105 (42%) cases were shown in a cross-sectional investigation, which studied young women in India [3]. The observed trends in LBP prevalence over the five years (2019-2023) reveal a dynamic and cyclical pattern, which may be influenced by a complex interplay of societal, healthcare, and global factors. The initial decline in LBP prevalence from 3767 (22.33%) cases in 2019 to 2762 (16.38%) cases in 2020 could be attributed to various factors, such as lifestyle modifications, changes in healthcare accessibility, or alterations in reporting practices. This decline aligns with studies that have documented the impact of the COVID-19 pandemic on population health, including reductions in physical activity levels and increased sedentary behavior, which can influence the prevalence of musculoskeletal conditions like LBP [21]. However, the subsequent resurgence in LBP prevalence, reaching 3817 (22.63%) cases by 2023, underscores the persistent and complex nature of this condition.

The analysis of BMI distribution among LBP patients reveals that 10328 (61.24%) cases are overweight or obese, while only 6228 (36.93%) cases have a normal BMI. This finding aligns with extensive research that has consistently identified excess body weight as a significant risk factor for LBP in diverse demographics [7]. The underlying mechanisms linking obesity and LBP include increased mechanical strain on the spine, altered biomechanics, and the pro-inflammatory state associated with excess adiposity [22]. Interestingly, the age-specific BMI patterns observed in this study, with a higher prevalence of overweight and obesity in middle-aged adults (39-59 years), are congruent with general population trends and the increased risk of LBP in this age group [23]. However, the high prevalence of LBP among young adults (18-38 years) with normal BMI suggests that factors beyond weight, such as physical activity levels, ergonomic factors, and psychological stress, may play a significant role in the development of LBP in this demographic [8,24,25]. Furthermore, the observed association reinforced the biopsychosocial interaction model between obesity and LBP, which is concordant with the previous findings by Malfliet et al. [26]. Therefore, the observed relationship between BMI and LBP represents a correlation rather than causation.

Clinical observations of comorbidities have shed light on managing LBP. This study showed that common issues among LBP patients include gastrointestinal disorders, hypertension, orthopedic disorders, and diabetes mellitus. The high rates of hypertension and diabetes align with findings from Asia, emphasizing the links between LBP, cardiovascular health, and overall well-being [27]. These connections highlight the need for integrated, multidisciplinary treatment strategies for chronic pain.

The predominance of gynecologists (20%) as top prescribers for LBP can be attributed to several interconnected factors evident in the epidemiological data. The significantly higher prevalence of LBP among females (67.71% vs. 32.29%, p < 0.001) aligns with previous studies showing that women experience more severe LBP symptoms than men [4]. The findings of the study revealed that LBP is particularly prevalent in women of reproductive age, with risk factors including multiple childbirths, type of delivery, and menopausal status [3]. This pattern suggests that women often report LBP during routine gynecological visits, making gynecologists their primary point of healthcare contact. The data from EMRs further demonstrates strong associations between LBP and female-specific health factors, with higher prevalence rates observed in postpartum women and those with multiple pregnancies [3]. This prescribing pattern underscores the need for integrated care approaches and potentially specialized LBP management training for gynecologists, given their significant role in women's healthcare delivery in India.

LBP management focuses mainly on NSAIDs and calcium supplements, which are effective for pain and inflammation, especially in older adults with osteoporosis. Opioid usage is low due to guidelines favoring non-opioid treatments. Calcium carbonate is commonly prescribed even though traditional guidelines do not emphasize it. Combination therapies with calcium and vitamin D3 were prescribed to 2024 (12%) of patients, pointing to changing pain management practices and the possible link between vitamin D and calcium deficiencies and chronic back pain. Further investigation of these treatment patterns may be necessary to establish the importance of calcium/vitamin D3 supplementation in LBP management [23]. The high prescribing pattern of calcium supplementation (26.56% of patients) in LBP management reflects multiple clinical considerations. The predominance of female patients (67.71%) in the study population, particularly those with postmenopausal status, aligns with established guidelines for calcium supplementation in women at risk for reduced bone density [23]. This prescribing pattern is further supported by the findings of this study that LBP frequently coexists with orthopedic conditions and endocrine disorders. While calcium supplementation is common, its specific role in LBP management requires further investigation through controlled trials.

Diclofenac is a widely used NSAID for managing pain and inflammation, either on its own or with other medications. Due to its proven effectiveness, it is often recommended as the first-line treatment for acute LBP [28]. Physicians often use a dual-therapy approach that combines NSAIDs and muscle relaxants to treat LBP. This combination helps reduce inflammation and muscle tension. Research by Friedman et al. indicates that it offers greater pain relief for acute lumbar pain compared to using either therapy alone [29]. Pregabalin is mainly used for neuropathic pain, but it is less common than NSAIDs and calcium supplements. It is not a first-line treatment for general LBP, but its use suggests a focus on neuropathic issues, consistent with guidelines for managing chronic LBP, as noted in reviews like Mathieson et al. [30]. Opioids, including tramadol with paracetamol, account for 632 of 16866 (3.75%) prescriptions due to dependence and side effects. Recent guidelines recommend a multimodal approach to LBP management, emphasizing NSAIDs and calcium/vitamin D supplements while advising caution with opioids and specific adjunct therapies like pregabalin.

Current prescribing patterns in India indicate a preference for calcium supplements, NSAIDs, and muscle relaxants, with careful use of opioids. This suggests a shift in LBP management. The high prescribing pattern of calcium supplementation in our study population suggests a practice pattern that may extend beyond current evidence-based guidelines for LBP management. While calcium supplementation plays a vital role in bone health maintenance, particularly in women at risk for osteoporosis [23], its specific role in LBP management requires further investigation through controlled trials. This prescribing pattern may reflect a preventive approach to skeletal health rather than a direct therapeutic strategy for LBP. There is a pressing need for standardized national guidelines that reflect the latest evidence-based practices and consider the complexity of the condition.

The study offers several notable strengths that enhance its contribution to LBP research in India. The large sample size of 16866 patients from geographically diverse regions provides robust generalizability, while the five-year study period (2019-2023) enables valuable insights into temporal trends, including the impact of the COVID-19 pandemic on LBP prevalence. The analysis of prescribing patterns across multiple specialties, combined with comprehensive demographic and anthropometric data, offers unique perspectives on real-world LBP management in the Indian healthcare system.

This study provides valuable insights into the epidemiology of LBP in India, but it is not without limitations. The retrospective cross-sectional nature of EMR data analysis restricted our ability to verify diagnosis accuracy, assess LBP progression and long-term outcomes, differentiate between acute, subacute, and chronic LBP cases, evaluate treatment effectiveness, disability progression, and quality of life changes. Another limitation of this study is the absence of data regarding non-pharmacological pain management strategies, including physical therapy interventions, exercise prescriptions, and ergonomic modifications, as the EMR system primarily captured medication-related data. Selection bias may have influenced our findings through the inclusion of only patients seeking medical care, potential socioeconomic barriers to healthcare access, and regional variations in EMR documentation practices. This gap in the documentation of comprehensive LBP management approaches limits our ability to evaluate the full spectrum of LBP care and its outcomes in the Indian healthcare setting. Further research with prospective designs and comprehensive data collection would be beneficial to corroborate and expand upon the findings of this study.

## Conclusions

This pan-Indian epidemiological cross-sectional study revealed LBP as a widespread health burden that significantly influenced patients' anthropometric parameters. The study identified key risk factors, including female gender, young adult age, and elevated BMI, which aligned with previous research findings. Calcium supplements, NSAIDs, and NSAID-muscle relaxant combinations were the most common treatment modalities, while opioid use was minimal. These findings provided valuable insights for healthcare policymakers to develop comprehensive LBP management strategies for the broader population.
